# Forecasting and prediction of scorpion sting cases in Biskra province, Algeria, using a seasonal autoregressive integrated moving average model

**DOI:** 10.4178/epih.e2016044

**Published:** 2016-10-14

**Authors:** Schehrazad Selmane, Mohamed L’Hadj

**Affiliations:** 1 L’IFORCE, Faculty of Mathematics, University of Sciences and Technology Houari Boumediene, Algiers, Algeria; 2 Health and Hospital Reform Services, Ministry of Health, Population and Hospital Reform, Algiers, Algeria

**Keywords:** Algeria, Biskra, Epidemiology, Forecasting, Scorpion stings, Time series

## Abstract

**OBJECTIVES:**

The aims of this study were to highlight some epidemiological aspects of scorpion envenomations, to analyse and interpret the available data for Biskra province, Algeria, and to develop a forecasting model for scorpion sting cases in Biskra province, which records the highest number of scorpion stings in Algeria.

**METHODS:**

In addition to analysing the epidemiological profile of scorpion stings that occurred throughout the year 2013, we used the Box-Jenkins approach to fit a seasonal autoregressive integrated moving average (SARIMA) model to the monthly recorded scorpion sting cases in Biskra from 2000 to 2012.

**RESULTS:**

The epidemiological analysis revealed that scorpion stings were reported continuously throughout the year, with peaks in the summer months. The most affected age group was 15 to 49 years old, with a male predominance. The most prone human body areas were the upper and lower limbs. The majority of cases (95.9%) were classified as mild envenomations. The time series analysis showed that a (5,1,0)×(0,1,1)_12_ SARIMA model offered the best fit to the scorpion sting surveillance data. This model was used to predict scorpion sting cases for the year 2013, and the fitted data showed considerable agreement with the actual data.

**CONCLUSIONS:**

SARIMA models are useful for monitoring scorpion sting cases, and provide an estimate of the variability to be expected in future scorpion sting cases. This knowledge is helpful in predicting whether an unusual situation is developing or not, and could therefore assist decision-makers in strengthening the province’s prevention and control measures and in initiating rapid response measures.

## INTRODUCTION

Algeria is faced with endemic scorpionism, with an annual average of around 50,000 scorpion sting cases. From 1991 to 2012, a total of 903,461 cases were recorded, of which 1996 resulted in death. Eighty-one percent of the country’s provinces are affected by this issue, but the incidence varies widely from the north to the south of the country; seven scorpion stings per 100,000 inhabitants are recorded in the northern provinces, whereas over 1,000 scorpion stings per 100,000 inhabitants are recorded in the southern provinces [1]. Algeria houses a diverse population of scorpions, and some provinces are seriously affected by scorpionism. More than 28 species have been catalogued in the country, of which four are dangerous to humans; namely, *Androctonus australis*, *Buthus tunetanus*, *Androctonus aeneas*, and *Androctonus crassicauda* [2]. The monitoring of scorpion envenomations is based on a passive system. The recording of scorpion sting cases, death investigations, and the standardisation of treatment are the main actions undertaken by health authorities. Neither analysis nor interpretation of the data has been undertaken to design intervention strategies. Given these concerns, the present study aims to highlight some epidemiological aspects of scorpion envenomations, to analyse and interpret the available data, and to develop a forecasting model for scorpion sting cases to estimate the variability in future cases in Biskra province, which records the highest number of scorpion stings nationally.

The geographical scope, diversity of ecosystems, and favourable climate of Biskra province represent a conducive environment for scorpion species, contributing to the endemicity of scorpion stings in this province. The yearly number of recorded scorpion stings in this province represents approximately 14% of the annually recorded cases on the national level [1]. Thus, Biskra province represents a rational choice to study in order to obtain an improved understanding of the epidemiology, incidence, lethality, circumstances, factors, and determinants of scorpion envenomations.

Multiple linear regression is the first mathematical approach to have been applied to scorpionism in the foundational study of Chowell et al. [3], who analysed the impact of climatological variables on scorpion sting incidence in humans in Colima state in Mexico. In line with this statistical approach, other studies have been performed in other affected regions of the world [4-6]. In this paper, in addition to an epidemiological survey, we performed a time series analysis based on the Box-Jenkins method [7]. To the best of our knowledge, this is the first time that this statistical approach has been applied to scorpionism in Algeria. In the first section, we briefly review this method, which represents a useful tool for monitoring scorpion sting cases, as well as providing estimates of future scorpion sting cases. These estimates can assist decision-makers in strengthening prevention and control measures, as well as initiating rapid response measures.

## MATERIALS AND METHODS

### Study area

The province of Biskra is located in central-eastern area of Algeria at 34°52´N and 5°45´E, at the gates of the Sahara. The province is made up of 33 municipalities distributed over 12 districts and stretches over a land size of approximately 20,986 km2. An estimated population of 820,766 people was recorded in 2013. Its Saharan climate is characterised by weak and irregular rainfall, high brightness, intense evaporation, and wide variations in temperature, reaching a monthly average of 34.8°C in July and a monthly average of 11.5°C in January. The dry period in the province lasts almost throughout the year [8].

### Scorpion sting data

The time unit used in the time series analysis was the month. The daily recorded sting cases from 2000 to 2013 were aggregated into months, generating 168 data points. The breakdown by sex of the affected individuals, anatomical sting site, age group, place, and the time slot used in the epidemiological survey for the year 2013 was provided by the Biskra Department of Public Health. No identification or personal information was revealed.

### Modelling method

A time series analysis based on the Box-Jenkins method models recorded cases over time and allows forecasts to be made of the expected numbers of recorded cases [9]. In adequate models, the time series should be stationary. Often, in practice, a time series is not stationary; that is, it exhibits non-stationary variance, a non-stationary mean, and periodic or seasonal components. To stabilise the variance, various transformations can be applied to each observation *X_t_* (*t* = 1,..., *n*), such as the logarithm, square root, or reciprocal. To stabilise the mean, an appropriate order of differences can render a non-stationary series a stationary one. If the series is observed to be seasonal with period S, Box and Jenkins proposed that the series could be modelled using a seasonal autoregressive integrated moving average (SARIMA) model. A (*p,d,q*)×(*P,D,Q*)*_s_* SARIMA model is defined by the following equation:

(1) $$A(L)\Phi (L^{S})\nabla^{d}\nabla_{S}^{D}X_{t}=B(L)\Theta (L^{S})\varepsilon_{t}$$

where *L* is the backward shift operator defined by $L^{k}X_{t}=X_{t-k},\nabla^{d}$ is the *d^th^* difference of *X_t_*, $\nabla_{S}^{D}$ is the *D^th^* seasonal difference operator with $\nabla_{S}=1-L^{S}$, and {ε_t_} is a white noise process. The polynomials *A*(*L*), *B*(*L*), Φ(*L*), AND Θ(*L*) are, respectively, the autoregressive (*AR*), the moving average (*MA*), the seasonal AR and the seasonal MA polynomials. The model must be stationary and invertible; that is, all roots of these polynomials have to be outside of the unit circle. Fitting a SARIMA model begins with the determination of the orders of *d, D, p, P, q, Q*, and *S*. In the supplementary material we describe how to determine these orders. The model equation (1) should be then estimated. It should be noted that based on the correlation structure, several possibilities for the values of *p* and *q* can be envisaged, thus producing several models. To test the model for goodness-of-fit, the residuals should be analysed. The residuals should be uncorrelated with a mean of 0 and follow a Gaussian distribution; moreover, the autocorrelations of the residuals should not be significantly different from 0 [7]. To select the best-fit model, we applied criteria such as the smallest Schwarz criterion, Akaike information criterion, standard error regression, the highest adjusted R^2^, the stationary and invertibility condition, and the white noise condition for residuals. Model equation estimations and analyses were performed using EViews (IHS EViews, Irvine, CA, USA) a statistical software offering access to powerful statistical, forecasting, and modelling tools through an innovative, easy-to-use object-oriented interface [10].

## RESULTS

**Figure 1.**
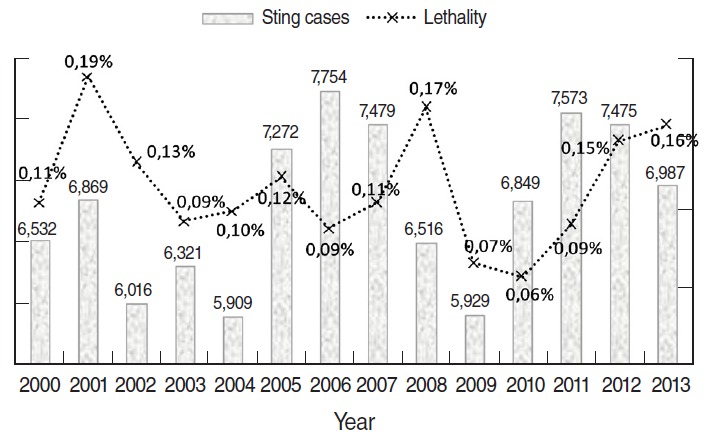
Yearly number of recorded scorpion sting cases and lethality in Biskra province.

**Figure 2.**
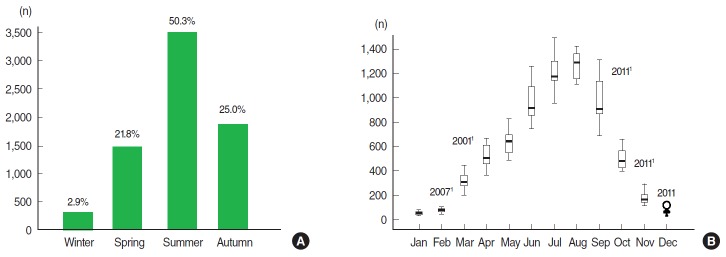
(A) Seasonal average and (B) Box plot with monthly average of recorded scorpion sting cases in Biskra province in the period 2000-2013. ^1^Correspond to outliers.

Among the 95,481 scorpion stings recorded by the Department of Public Health of Biskra province between 2000 and 2013, 112 cases resulted in deaths. The global annual number of recorded scorpion stings and lethality are plotted in Figure 1. The lethality corresponds to the ratio of the deaths due to scorpion envenomations over the number of people stung during the same period and is expressed as a percentage. The highest annual number of recorded scorpion stings occurred in 2006, with 7,754 cases, and the lowest occurred in 2004, with 5,909 cases. The highest number of deaths was recorded in 2001, with 13 deaths, followed by 2008, 2012, and 2013 with 11 deaths. Stings were recorded throughout the year, with major peaks in the summer months. Half of the stings were recorded during summer period, followed by the autumn period with a quarter of the stings, and in last place, the winter period (Figure 2A). The mean monthly distribution of scorpion sting cases was as follows: 9.3% in May, 14.1% in June, 17.6% in July, 18.7% in August, and 14.4% in September (Figure 2B). The monthly maximum reported scorpion sting cases occurred in July 2005 with 1,490 cases, followed by August 2008 with 1,420 cases. Outliers were observed in March 2001 and in October, November, and December 2011, with 659, 1,110, 401, and 122 recorded cases, respectively, which were almost twice as high as the corresponding monthly means. During 2011, scorpion collections were not as fruitful as those conducted in previous years, meaning that date picking, which begins in October in this region and leads to increased accidents, could have involved an abnormal number of scorpion stings during that year.

### Geographical distribution of scorpion envenomation

**Figure 3.**
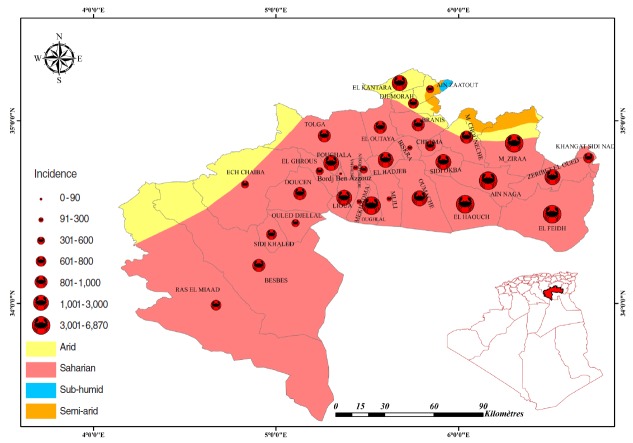
Geographical distribution of the incidence of scorpion stings in Biskra province.

The population size for each of the 33 municipalities was estimated from the 2008 census [11]. The annual number of scorpion stings was normalized to the total population and expressed as cases per 100,000 inhabitants. The geographical distribution for the year 2013 of scorpion sting incidence per 100,000 inhabitants was mapped using MapInfo 11 (Figure 3). All municipalities were affected by scorpionism; Mziraa was ranked first with 6,868 cases per 100,000 inhabitants, followed by Ain Naga, with 4,529 cases per 100,000 inhabitants.

### Epidemiological survey

During the year 2013, 6,987 scorpion sting cases were recorded; males were more affected than females, with 62% of the recorded sting cases. Among the 11 recorded deaths, six were males. Deaths were observed in all age groups and in both sexes. An examination of death certificates from 2000 to 2013, provided by the Department of Public Health of Biskra, reveals that most deaths occurred in a hospital centre, most of the deceased were rural residents, and the majority of fatal cases were evaluated as moderate or severe envenomations at the time of their first medical examinations.

The extremities of the upper limbs (3,363 cases) and the extremities of the lower limbs (3,124 cases) were the parts of the human body most prone to scorpion stings, followed by the trunk (319 cases) and the head and neck (181 cases).

The age group most affected by scorpion stings was the age group of 15 to 49 years (66% of stung people), followed by children less than 14 years old (19% of cases). Mortality was higher in children than in adults, with seven of the 11 deaths occurring among children.

Of the sting cases, 46% occurred within dwellings, of which 54% occurred in females, whereas outside of dwellings, males were the most vulnerable, with 76% of sting cases. Scorpion stings took place throughout the day, with a peak between 6:00 a.m. and 12:00 p.m. (41% of cases), followed by 6:00 p.m. to 12:00 a.m. (36% of cases), and 12:00 a.m. to 6:00 a.m. and 12:00 p.m. to 6:00 p.m., with 12% and 11% of cases, respectively.

The clinical manifestations of scorpion envenomation fall into three grades: grade I for local events, grade II for mild systemic symptoms, and grade III for life-threatening envenomations [12]. For the year 2013, 95.9% of cases were graded as mild, 3.4% of cases were graded as moderate, and 0.7% of cases were graded as severe. A total of 6,214 antivenom vials were used, corresponding to coverage of 89% of the cases. The corresponding medical costs were estimated as 73,478 USD, resulting in a serious financial burden for the province.

### Statistical modelling output

**Figure 4.**
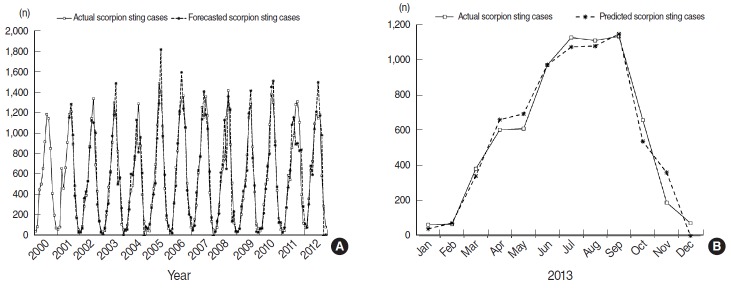
(A) Recorded number of scorpion sting cases between 2000 and 2012 and the number of cases estimated by the (5,1,0) × (0,1,1)_12_ seasonal autoregressive integrated moving average model. (B) The predicted and actual number of scorpion sting cases for the year 2013.

The data were split into two periods; the data from January 2000 to December 2012 were used for model development, and the data from January 2013 to December 2013 were used for validating the model and forecasts. We started the analysis by applying the Box-Jenkins modelling steps to the monthly scorpion sting data once stationary conditions were achieved. To achieve stationary conditions, one differencing step and a 12-month differencing of the original data were necessary. Moreover, the autocorrelation and partial autocorrelation functions allowed us to identify possible values for the remaining orders. After several trials, the (5,1,0)×(0,1,1)_12_ SARIMA model, displayed in Appendix 1, was selected as appropriate to the scorpion sting time series data; all of the features of the best model were fulfilled. Consequently, forecasting of the time series could be made on the basis of this model. The simulated scorpion sting cases for the 2000 to 2012 period closely approximated the recorded data (Figure 4A), with a very strong correlation (Pearson product-moment correlation coefficient, *r*=0.939). The model was then used to predict the monthly scorpion sting cases from January 2013 to December 2013. The fitted data showed considerable agreement with the actual data, as shown in Figure 4B, and the correlation between actual and fitted cases was very strong (*r*=0.984). All these conclusions attest to the adequacy of the model.

**Figure 5.**
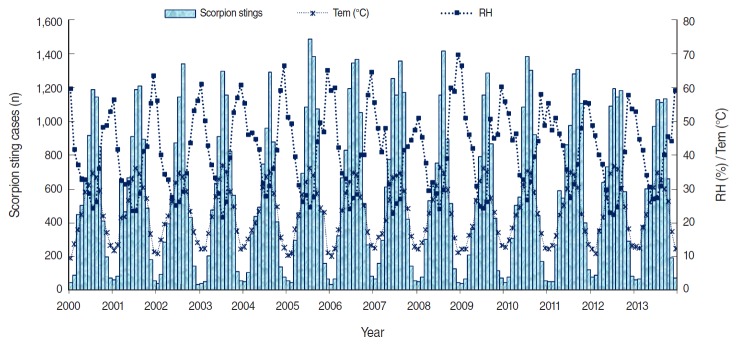
Time series of monthly recorded scorpion sting cases with monthly average temperatures (Tem) and monthly average relative humidity (RH) values in Biskra province, 2000-2013.

Climatological factors may be assumed to be of great influence on scorpion distribution and activity. We therefore examined the relationship between monthly sting cases and some climatological variables. Scorpion sting cases were strongly positively correlated with temperature (*r*=0.954), highly negatively correlated with relative humidity (*r*=-0.803), and weakly correlated with precipitation and wind speed. The time series of monthly sting cases, monthly mean temperatures, and monthly relative humidity values plotted in Figure 5 show the relationships over time between these variables; monthly scorpion sting cases followed the same trend as temperature, and an opposite trend to relative humidity. An extension of SARIMA modelling in an attempt to predict scorpion sting cases using these climatological variables was undertaken, but no best model was found.

## DISCUSSION

Envenomations by scorpion stings are an important health concern in Biskra province. Over a period of 14 years from 2000 to 2013, the province was ranked first in scorpion sting accidents, with 95,481 scorpion sting cases recorded. The annual average incidence is 972 stings per 100,000 inhabitants, which is significantly higher than the national rate (estimated to be 152 stings per 100,000 inhabitants) [1]. Approximately half of scorpion stings occur in summer, with the most recorded cases in August (18.7%) followed by July (17.6%), corresponding to the hottest and driest months of the year. This is in accordance with studies performed in other regions affected by scorpionism [5,13,14]. All municipalities of the province are affected by scorpionism at different levels, and most cases have been recorded in rural areas.

The epidemiological analysis showed that males were stung much more often than females (62% vs. 38%); a similar trend has also been observed on the national level (57% vs. 43%) [15] and in findings reported from other countries [14,16]. The age group of 15 to 49 years old was the most commonly affected, including 66% of stung people. Various studies have shown diverse age distributions for scorpion sting cases; the findings reported in [13,17] are not similar to our conclusions. This discrepancy therefore corroborates the conclusions made by Chippaux & Goyffon [18] on geographical variation in epidemiological indicators.

Our epidemiological investigation established that humans were responsible for scorpion accidents. The parts of the human body most affected by scorpion stings were the extremities of the upper and lower limbs, with 93% of reported victims, a frequency not significantly different from the results reported by Rafizadeh et al. [13]. The fact that the ends of the upper limbs and lower limbs were the most affected parts of the human body shows the great responsibility of humans for these accidents through negligence, ignorance, or both; a public awareness program could reduce the incidence of stings significantly only if people are involved in the development and implementation of such programs. Unplanned construction in rural areas is creating problems, as people are building directly on top of scorpion shelters. Furthermore, detritus litters the streets and public spaces, thereby promoting outbreaks of cockroaches, flies, and other arthropods, thus providing plentiful prey for scorpions. Thus, the scorpion finds a supportive environment with abundant food safely and, above all, without the need to use its venom. In this way, humans help in the reduction of consumption of scorpions by scorpions and thereby facilitate the proliferation of scorpions. Therefore, to counter the proliferation of scorpions and incidents involving scorpions, it is highly recommended to review the strategies in place and to go beyond mere informational campaigns. The involvement of the population in the search for solutions and decision-making related to this issue is needed, and this must take place over the long term, with due consideration of environmental, social, and economic factors.

The first studies conducted on the identification and geographical distribution of scorpion species in Algeria were carried out by Vachon [2]. Recent studies have been performed in Belezma National Park [19]. Given the high rates of mortality and scorpion sting accidents that are recorded each year in Biskra province, research including zoogeography and identification of the species involved in envenomations is suggested; this will certainly reinforce the surveillance system and will help devise appropriate strategies to contain the scorpion envenomation problem in this province.

This study highlighted some epidemiological aspects of envenomations by scorpion stings in Biskra province. In addition to analysis and interpretation of the available data, the SARIMA method was shown to be useful for monitoring scorpion sting cases and providing estimates of future scorpion sting cases. This knowledge is helpful for predicting whether an unusual situation is developing. It could therefore assist decision-makers in having clearer ideas about strengthening the province’s prevention and control measures, as well as facilitate the initiation of rapid response measures. Consequently, the integration of forecasting methods into surveillance systems is becoming necessary to assist public health services in containing envenomations by scorpions and ensuring a state of preparedness.

## Appendix 1.

Model estimation
