# Supplementary material for: Forecasting and prediction of scorpion sting cases in Biskra province, Algeria, using a seasonal autoregressive integrated moving average model
Source: Epidemiol Health. 2016 Oct 14;38:e2016044. doi: 10.4178/epih.e2016044 (PMC5177803; doi:10.4178/epih.e2016044)
Supplement: Supplementary file 1 [file epih-38-e2016044-app1.pdf]

Appendix 1. Model estimation

Variable	Coefficient	SE	t-statistic	Probability
AR(1)	-0.441	0.086	-5.136681	<0.001
AR(2)	-0.388	0.089	-4.348525	<0.001
AR(3)	-0.317	0.088	-3.597259	<0.001
AR(4)	-0.326	0.086	-3.797495	<0.001
AR(5)	-0.152	0.083	-1.814554	0.07
MA(12)	-0.905	0.020	-45.13634	<0.001
R-squared	0.579	Mean dependent Var		-0.239
Adjusted R-squared	0.564	SD dependent Var		168.940
SE of regression	111.602	AIC		12.310
Sum squared residual	1,644,056.000	Schwarz criterion		12.437
Log likelihood	-843.408	Hannan-Quinn criterion		12.362
Durbin-Watson statistic	2.022			
Inverted AR roots	0.42-0.66i	0.42+0.66i	-0.38-0.56i	-0.38+0.56i
	-0.54			
Inverted MA roots	0.99	0.86+0.50i	0.86-0.50i	0.50+0.86i
	0.50-0.86i	0.00+0.99i	-0.00-0.99i	-0.50+0.86i
	-0.50-0.86i	-0.86+0.50i	-0.86-0.50i	-0.99
Dependent Var: X<br>Method: Least squares<br>Sample (adjusted): Jul 2001 to Dec 2012<br>Included observations: 138 after adjustments<br>Convergence achieved after 6 iterations				

SE, standard error; AR, autoregressive; MA, moving average; SD, standard deviation; AIC, Akaike information criterion; Var, Variable.
